# Acute Kidney Injury among Patients Visiting the Nephrology Unit in a Tertiary Care Centre: A Descriptive Cross-sectional Study

**DOI:** 10.31729/jnma.7916

**Published:** 2022-12-31

**Authors:** Krishna Kumar Agrawaal, Prabesh Raghubanshi, Anjana Bohaju, Jiya Acharya

**Affiliations:** 1 Department of Internal Medicine, Nephrology Unit, Universal College of Medical Sciences, Siddhartha Nagar, Bhairahawa, Nepal; 2 Kathmandu Medical College and Teaching Hospital, Sinamangal, Kathmandu, Nepal

**Keywords:** *acute kidney injury*, *mortality*, *renal replacement therapy*

## Abstract

**Introduction:**

Acute kidney injury is an abrupt decline in renal function often associated with a decrease in urine output. It is the leading cause of in-hospital mortality worldwide with prolonged hospital stays, the requirement of mechanical ventilation and short-term dialysis. The aim of the study was to find out the prevalence of acute kidney injury among patients visiting the Nephrology unit in a tertiary care centre.

**Methods:**

This descriptive cross-sectional study was done among patients presented to the Nephrology Unit of the Department of Internal Medicine in a tertiary centre from 9 February 2022 to 21 October 2022. Ethical approval was taken from Institutional Review Committee (Reference number: UCMS/ IRC/047/22). Data was collected from hospital records and the outcome was recorded in terms of in-hospital mortality and the requirement for renal replacement therapy. Convenience sampling was done. Point estimate and 95% Confidence Interval were calculated.

**Results:**

Among 1848 patients, 113 (6.12%) (5.03-7.21, 95% Confidence Interval) had acute kidney injury. About 38 (32.75%) required inotropes whereas 10 (8.85%) required mechanical ventilation. In-hospital all-cause mortality was seen in 14 (12.39%) of the study population and 20 (17.70%) of the study population required renal replacement therapy. The most common cause of acute kidney injury was infection pneumonia followed by acute gastrointestinal infections were the most common infective aetiology.

**Conclusions:**

The prevalence of acute kidney injury was found to be lower than the studies done in similar settings. It is common in patients admitted with infection. It is responsible for in-hospital mortality.

## INTRODUCTION

Acute kidney injury (AKI) is an abrupt decline in renal function often associated with a decrease in urine output.^1^ AKI is a leading cause of in-hospital mortality worldwide.^2^ It is related to a prolonged hospital stay and mechanical ventilation with severe forms requiring short-term dialysis.^3^

Improving Global Outcomes (KDIGO) group has published a consensus definition and classification for AKI.^4^ KDIGO covers both acute kidney injury network (AKIN) and Risk, Injury, Failure, Loss of Kidney Function, and End-Stage Kidney Disease (RIFLE) criteria, taking into account changes in creatinine within 48 hours or a decline in glomerular filtration rate (GFR) over 7 days.^5^ Etiology and incidence of AKI differ between high-income and low-to-middle-income countries.^6^ Epidemiologic studies of AKI are sparse, making incidence, prevalence and particularly outcomes difficult to compare.^7^

The aim of the study was to find out the prevalence of acute kidney injury among patients visiting the Nephrology unit in a tertiary care centre.

## METHODS

This descriptive cross-sectional study was conducted from 9 February 2022 to 21 October 2022 in the Nephrology Unit, Department of Internal Medicine at Universal College of Medical Sciences (UCMS). Ethical approval was taken from Institutional Review Committee (Reference number: UCMS/IRC/047/22). Data was collected from hospital records and the outcome was recorded in terms of in-hospital mortality and the requirement for renal replacement therapy. Patients admitted under the Nephrology Unit in the Department of Internal Medicine were included in the study. Patients with incomplete hospital data, underlying chronic kidney disease, glomerular disease and structural damage to the kidneys were excluded from the study. Convenience sampling was done and the sample size was calculated using the formula:


$$\begin{array}{ll} \text{n} & ={\text{Z}}^{2}\times \frac{\text{p}\times \text{q}}{{\text{e}}^{\text{2}}}\\ & =1.96^{2}\times \frac{0.08\times 0.92}{0.02^{2}}\\ & =707 \end{array}$$

Where,

n = minimum required sample size,
Z = 1.96 at 95% Confidence Interval (CI),
p = prevalence of patients with AKI taken from previous study, 8%^8^
q = I-P
e = margin of error, 2%

The calculated sample size was found to be 707. On doubling, the sample size becomes 1414. However, we took 1848 samples.

Patients were classified based on KDIGO staging for AKI^8^ and the outcome was recorded in terms of inhospital all-cause mortality and requirement of renal replacement therapy (RRT). Patients were provided with either conventional hemodialysis or sustained low-efficiency dialysis. Data were analysed using IBM SPSS Statistics version 17.0. Point estimate and 95% CI were calculated.

## RESULTS

Among 1848 patients, 113 (6.12%) (5.03-7.21, 95% Confidence Interval) had acute kidney injury. The mean age was 47.09±16.86 years with a male: female ratio of 1.21:1 (Table 1).

**Table 1:** Socio-demographic data population (n= 113)

Characteristics		n (%)
**Age group (in years)**	18-39	43 (38.05)
	40-59	37 (32.74)
	≥60	33 (29.21)
**Gender**	Male	62 (54.87)
	Female	51 (45.13)
**Religion**	Hindu	101 (89.38)
	Muslim	10 (8.85)
	Buddhist	2 (1.77)

Ninety-one (80.53%) patients had infective causes as the aetiology of acute kidney injury and 1 (0.89%) had an electric shock as the aetiology (Table 2).

**Table 2:** Aetiology of AKI of the study population (n= 113).

Aetiology of AKI	n (%)
Infective causes	91 (80.53)
Chronic liver disease	8 (7.08)
Acute pancreatitis	6 (5.31)
Wasp bite	4 (3.54)
Diabetic ketoacidosis	3 (2.65)
Electric shock	1 (0.89)

**Figure 1:**
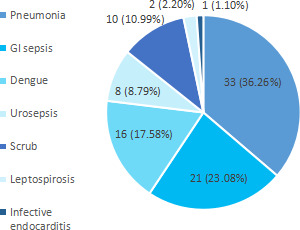
Infective causes of AKI of the study population (n= 91).

Among infective causes, 33 (36.26%) had pneumonia followed by GI sepsis in 21 (23.08%) (Figure 1).

Among the comorbidities, 35 (31.00%) had diabetes mellitus, 36 (31.90%) had hypertension and 5 (4.40%) had ischemic heart disease. The mean serum creatinine level at admission was 3.50±2.24 mg/dl and the mean estimated glomerular filtration rate on admission calculated using chronic kidney disease epidemiology collaboration equation was 35.04±20.36 ml/min/1.73 m^2^. The average duration of hospital stay was 6.27±2.56 days.

Forty-seven (41.59%) of the patients had a KDIGO stage 3 whereas 21 (18.58%) had stage 1 (Table 3).

**Table 3:** KDIGO staging of AKI in study population (n= 113).

KDIGO staging of AKI	n (%)
Stage 1	21 (18.58)
Stage 2	45 (39.82)
Stage 3	47 (41.59)

About 38 (33.62%) required inotropes whereas 10 (8.85%) required mechanical ventilation. In-hospital all-cause mortality was seen in 14 (12.40%) of the study population. About 20 (17%) of the study population required RRT.

## DISCUSSION

Among 1848 patients, 113 (6.12%) had acute kidney injury. Our study of 113 patients with AKI showed infection as the major contributor seen in more than two third of the study population which is similar to a study at Bir Hospital.^9^ Among the infections, pneumonia (36%) followed by acute gastrointestinal (GI) infections (23%) was the major cause leading to AKI whereas in another study the leading cause of AKI was GI infections.^10,11^ This implies that pneumonia as a cause of AKI is increasing as shown by another study in 2019.^12^ Mean serum creatinine at admission was 3.50±2.24 mg/dl which was similar to a study. About 20 (17%) of the study population required RRT implying that more than 80% were managed conservatively which is in accordance with the study from Nepal Medical College.^13^

Our study showed that 41% of the study population had KDIGO Stage III AKI and nearly half of them required RRT. Nearly 30% required ionotropic support to maintain mean arterial pressure and 10% required Mechanical ventilation. Mortality in patients with AKI was 12.40% which is similar to a study.^14^ Thus, our study had similar findings to previous studies and it used the revised consensus classification of AKI based on KDIGO guidelines.^8^

The limitation of our study is that serum creatinine in itself is an imperfect marker of kidney function. It is affected by the use of drugs, and muscle mass and also it lags behind the fall in GFR thus early detection is not possible. The use of novel biomarkers for AKI was not feasible in our setting.

## CONCLUSIONS

The prevalence of acute kidney injury was found to be lower than the studies done in similar settings. Acute kidney injury is common in patients admitted with infection. It is responsible for in hospital mortality. Majority of the patients can be managed conservatively though few require short term dialysis.
